# Case report of congenital asplenia presenting with *Haemophilus influenzae* type a (Hia) sepsis: an emerging pediatric infection in Minnesota

**DOI:** 10.1186/s12879-019-4572-4

**Published:** 2019-11-08

**Authors:** Tiffany Albrecht, Kristina Poss, Satja Issaranggoon Na Ayuthaya, Lori Triden, Katherine L. Schleiss, Mark R. Schleiss

**Affiliations:** 1 Department of Pediatrics, University of Minnesota Masonic Children’s Hospital, 2450 Riverside Ave, Minneapolis, MN 55454 USA; 2 University of Minnesota Medical School, 420 Delaware Street SE, Minneapolis, MN 55455 USA; 3 Present address: Department of Pediatrics, Lincoln Medical Center, 234 E 149th Street, The Bronx, NY 10451 USA; 4 Present address: Children’s National Medical Center, 111 Michigan Ave NW, Washington, DC 20010 USA; 5 Minnesota Department of Health, Infectious Diseases, Epidemiology and Control Division, 625 Robert Street N, Saint Paul, MN 55155 USA

**Keywords:** Haemophilus influenze type A, Haemophilus influenze type B, Asplenia, Hib vaccines, Isolated congenital asplenia, *Haemophilus influenzae* vaccines, Septic arthritis

## Abstract

**Background:**

In the pre-vaccine era, invasive disease with *Haemophilus influenzae*, type b (Hib) commonly presented with osteoarticular involvement. *Haemophilus influenzae*, type a (Hia) sepsis is a rare but emerging problem in recent years. Here, we report a case of sepsis with concomitant osteoarthritis due to Hia that was the presenting infectious disease manifestation of isolated asplenia in a young child. This unique observation adds to our understanding of sepsis and asplenia in children.

**Case presentation:**

A five-year-old girl developed acute Hia bacteremia and sepsis. The patient developed arthritis shortly after onset of septic shock. Arthrocentesis was culture-negative, but given the difficulty differentiating between septic and reactive arthritis, prolonged antibiotic administration was provided for presumed osteoarticular infection, and the patient had an uneventful recovery. The finding of Howell-Jolly bodies on blood smear at the time of presentation prompted an evaluation that revealed isolated congenital asplenia. Evaluation for known genetic causes of asplenia was unrevealing. Investigation by the Minnesota Department of Health revealed an emergence of Hia infections over the past 5 years, particularly in children with an American Indian background.

**Conclusions:**

Hia is an important pathogen in the differential diagnosis of invasive bacterial infections in children and shares overlap in clinical presentation and pathogenesis with Hib. Invasive Hia disease can be a presenting manifestation of asplenia in children. Hia is an emerging pathogen in American Indian children.

## Background

Invasive infections caused by *Haemophilus influenzae* type b (Hib) were once common in clinical pediatric practice, and included bacteremia, meningitis, pneumonia, skin and soft tissue infections, and bone and joint infections [1]. With the advent of conjugate Hib vaccine, invasive infections with this organism have become very rare in children. However, recent years have seen an emergence in invasive disease due to other non-type b serotypes, in particular type a. An increased risk of disease due to *Haemophilus influenzae* type a (Hia) has been described in American Indian children [2]. We describe in this case report a case of Hia sepsis in a previously healthy, five-year-old child. Isolated congenital asplenia (ICA) was an unexpected discovery at the time of the child’s presentation. Several aspects of this case of Hia sepsis are unique, including the presentation of Hia sepsis as the initial manifestation of ICA. This case was also noted against the backdrop of a recently recognized emergence of invasive Hia in Minnesota in the past 5 years. This case report adds to the medical literature, both by alerting clinicians to the fact that Hia is an emerging infection which should be included in the differential diagnosis for serious bacterial infection in children, and by demonstrating that this infection can be a presenting manifestation of congenital isolated asplenia.

## Case presentation

A five-year-old, fully vaccinated, American Indian female presented to a rural community hospital in Minnesota with a 4-day history of vomiting and diarrhea, followed by the development of fevers and progressive lethargy. A clinical diagnosis of sepsis was made. Blood cultures were obtained in the local emergency department, and she was commenced on empiric ceftriaxone (75 mg/kg dose) and vancomycin (15 mg/kg dose) therapy prior to her urgent transfer to the University of Minnesota Masonic Children’s Hospital. The main symptoms of the patient and the important clinical findings are described below. Upon arrival, she was febrile to 38.1 °C with a heart rate of 180, respiratory rate of 48, and blood pressure of 81/46. Physical exam was notable for lethargy, dry mucous membranes, and diffuse abdominal tenderness. Laboratory evaluation demonstrated a serum lactic acid of 8.8 mmol/L, creatinine of 1.05 mg/dL, leukocyte count of 10,200 cells per μL, hemoglobin 11.2 g/dL, and platelet count of 15,000 cells per μL. Inflammatory markers were elevated, with a C-reactive protein of 286 mg/L and a serum procalcitonin of > 200 ng/mL. Cerebrospinal fluid analysis demonstrated a leukocyte count of 3 cells per μL, glucose of 58 mg/dL, and protein level of 35 mg/dL. No organisms were noted on gram-stain. A peripheral smear was examined and demonstrated the presence of Howell-Jolly bodies. An abdominal ultrasound obtained to evaluate for intra-abdominal infection revealed complete anatomic asplenia. An echocardiogram demonstrated no cardiac anomalies. Within 24 h of admission, a blood culture drawn on initial presentation at the community hospital grew a gram-negative coccobacillary organism that was subsequently identified as *H. influenzae*. Antibiotic therapy was modified to ceftriaxone monotherapy, and ongoing clinical improvement was observed. The bacterial culture was subsequently identified by the Minnesota Department of Health as *H. influenzae*, serotype a, by use of a serotype-specific polymerase chain reaction assay, employing appropriate controls.

**Fig. 1:**
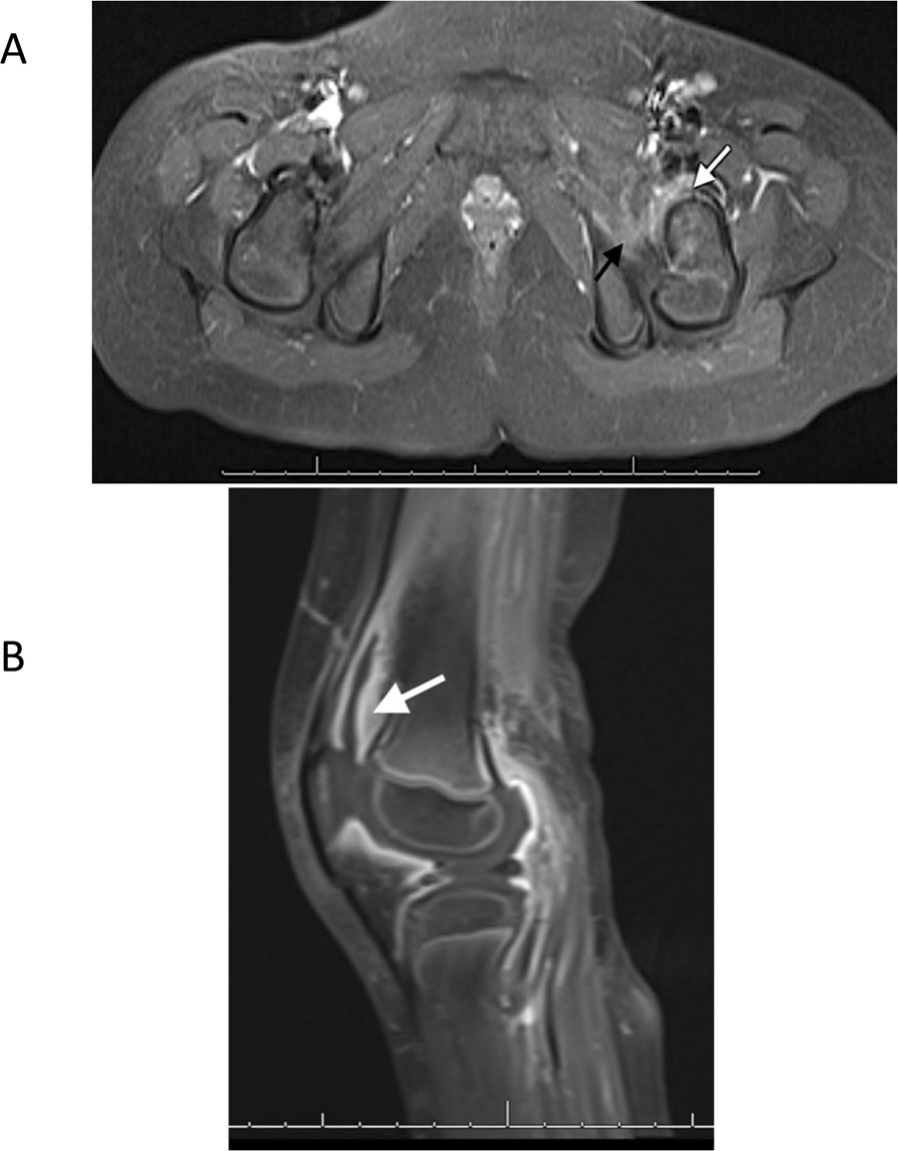
Radiographic findings of reactive arthritis following Hia sepsis. **a**, Left-sided joint effusion with synovial enhancement noted in the left hip (white arrow). In addition, asymmetric myositis involving the lateral fibers of the left gluteus medius/minimus and the proximal/deep fibers of the vastus intermedius and vastus lateralis musculature is also observed (black arrow) **b**, Enhancement and soft tissue swelling was noted by MRI of left knee with synovial thickening (white arrow) and effusion present

On hospital day 3, the patient was noted to have left knee and elbow pain. The main diagnoses, interventions and outcomes are described below. First, a knee radiograph demonstrated a large joint effusion. Analysis of aspirated knee synovial fluid was remarkable for a leukocyte count of 16,750 cells per μL (96% neutrophils), but no organisms were noted on gram-stain. Left hip pain developed and a technetium ^99^m bone scan demonstrated asymmetric signal increase in the left proximal femur, while an MRI revealed joint effusion and myositis without evidence of osteomyelitis (Fig. 1). Irrigation and debridement of the left hip was performed, but cultures from synovial fluid were also negative, possibly due to the previous antibiotic therapy.

Next, in light of the newly diagnosed isolated congenital asplenia, further investigation of medical and family history was pursued. This more detailed history confirmed that the patient was previously healthy, with no history of severe or frequent infections. The family was not aware of any other relatives with asplenia. Next generation sequencing for the ribosomal protein SA (RPSA) gene associated with isolated anatomic asplenia was obtained [3], but did not demonstrate any mutations. An immunological evaluation revealed protective antibody level to Hib (14.7 μg/mL; >1.0 μg/mL = protective level) and most serotypes of a 13-valent pneumococcal conjugate vaccine. The patient was discharged home with a peripherally-inserted central catheter and intravenous ceftriaxone was extended to a total 6 weeks course of therapy, due to her slow clinical recovery that included persistent limping and limited range-of-motion of her left knee, noted at the 3-week follow up visit. After completion of intravenous antibiotics, she was doing well with restored function of her left elbow, knee and hip and she had undetectable C-reactive protein and procalcitonin levels. She was later transitioned to daily amoxicillin prophylaxis for her asplenia.

## Discussion and conclusion

**Fig. 2:**
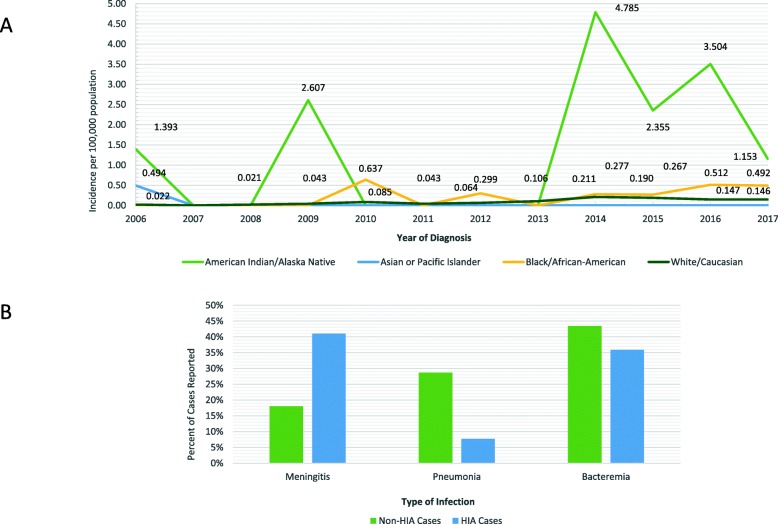
Epidemiology of Hia disease in Minnesota. **a**, Invasive Hia disease by race in Minnesota, 2006–2017. An emergence of Hia disease is noted in American Indian children, particularly since 2013, peaking at ~ 4.8 cases/100,000 population in 2014. **b**, disease characteristics of invasive Hia disease compared to non-Hia cases in children under 5 years of age. Hia cases in Minnesota noted to have ~ 2-fold increased risk of presenting as meningitis compared to the non-Hia cases of invasive *Haemophilus influenzae* disease

Invasive disease due to Hib includes sepsis, septic arthritis, pericarditis, osteomyelitis, and meningitis. Hib invasive disease was once common in children, prior to development of an effective vaccine: indeed, approximately 20,000 instances of invasive Hib disease occurred annually in the United States in the pre-vaccine era, affecting about 1 in 200 children under 5 years of age [1, 4]. In the post-conjugate Hib vaccine era, the emergence of serious infections caused by non-type b strains, particularly Hia strains, has been observed [5]. These infections have been observed with increased frequency recently in Minnesota (Fig. 2a), particularly in children of American Indian ancestry [2, 6–8]. The emergence of invasive Hia has been most striking in children under the age of 5 years, and this species is today more likely than non-type a strains to be associated with bacterial meningitis (Fig. 2b). The child we describe in the current case report was an American Indian of Ojibwe ancestry, and was from central Minnesota. Both observations were of interest in light of recent data reported by the Minnesota Department of Health [8] that Hia cases were more likely to be observed in American Indian children (*p* < 0.001) and in children living outside of the Twin Cities (Minneapolis-St. Paul) area (*p* = 0.011).

A surprising finding in our case was the discovery of Howell-Jolly bodies on a peripheral blood smear at the time of our patient’s initial presentation in septic shock. This was followed by ultrasonographic confirmation of asplenia. The absence of any cardiovascular abnormalities (demonstrated by a normal echocardiogram) ruled out heterotaxy syndromes (such as Ivemark syndrome) associated with asplenia [9], resulting in the diagnosis of ICA. Sepsis is often the first presenting sign of ICA; 78% of cases in one review presented with severe invasive bacterial infection [10], most commonly caused by *S. pneumoniae* and, less commonly, by *Haemophilus influenzae*. The genetic basis of ICA is unknown. It has been proposed that sporadic cases are associated with de novo mutations in genes important in spleen development [11]. For autosomal dominant familial cases, a recent study suggests mutations in ribosomal protein SA (*RPSA*), which encodes ribosomal subunit protein SA, may be implicated [3], although no *RPSA* mutation was identified in the patient described in this report. Another proposed genetic mechanism is through mutations in *Hox 11*, since transgenic mice with targeted knock-out of this gene have asplenia [11].

Remarkably, in spite of her ICA, our patient had no previous history of other serious bacterial infections. Only a handful of cases of Hia sepsis have been previously identified in children with impaired or absent spleen function. An Israeli study of serious bacterial infections in 34 children with functional hyposplenism demonstrated two cases of Hia sepsis [12]. A case of fulminant Hia sepsis, presenting with bacteremia, septic arthritis and meningitis, was recently described in a four-year-old child with asplenia and associated heterotaxy [13]. The current case described in this report is, to our knowledge, the first case report of bacterial sepsis due to Hia presenting as the initial manifestation of ICA. Since Hia is an encapsulated organism, it should be added to the list of pathogens known to be associated with invasive bacterial infection in the setting of hyposplenism, particularly in light of its recent documented emergence both in the general population and in specific high-risk populations.

The rationale for our conclusions in this case stems from observations regarding the recent emergence of invasive Hia disease in children, suggesting that this strain may be filling an ecological niche left by the eradication of Hib nasopharyngeal carriage, conferred by widespread use of the highly successful Hib vaccine. However, whether or not the emergence of Hia represents a bona fide “strain replacement” of Hib remains debatable [14]. Interestingly, it has been noted that the capsular composition of Hia is most similar (among the encapsulated strains) to that of Hib [13], and its capsule is the most resistant to complement-mediated lysis of all of the non-type b strains [15, 16]. Notably, the emergence of Hia as an invasive pediatric pathogen has promoted discussion about whether development of a vaccine is required as a public health measure [7], particularly for American Indian children, and for residents (especially of aboriginal descent) in provinces of northern Canada [17] in whom the prevalence of infection is also increased. In Minnesota, we have observed an increase in *Haemophilus influenzae* invasive disease incidence and specifically in Hia cases from 2006 to 2017 (Fig. 2a). Hia cases have been noted to be more likely encountered in American Indian children (< 5 years), residents of greater Minnesota (as opposed to the Twin Cities), and in patients that present with meningitis.

In summary, what are the main “take-away” lessons of our case? First, our case illustrates that clinicians should be mindful of the emergence of Hia as a cause of invasive infection in children, particularly those of American Indian background. We recommend that all encapsulated strains of *H. influenzae* isolated from sterile body sites should undergo serotyping, and be reported to local health departments. A second “take-away” lesson is that consideration should be given to investigation of possible underlying immune deficiencies, in particular hyposplenism, in any child with Hia sepsis. These detailed descriptions are strengths of this case report, but an obvious limitation is the paucity of published data on the range of manifestations of invasive Hia disease. Data reported in this case report should help address this deficiency. Future surveillance will be essential in developing approaches for development and possible implementation of candidate Hia subunit vaccines [18] as a prevention strategy for this emerging infection.

## Data Availability

All data and materials are available from the corresponding author.

## Abbreviations

Hia: *Haemophilus influenzae*, type A
Hib: *Haemophilus influenzae*, type B
ICA: Isolated congenital asplenia
MDH: Minnesota Department of Health
MRI: Magnetic resonance imaging
RPSA: Ribosomal protein SA

## References

[1] Kline MR, Blaney SM, Giardino AP, et al. Chapter 258, *Haemophilus influenzae*. In: Rudolph’s pediatrics. 23rd ed. New York: McGraw-Hill education / medical. 2018.

[2] Millar EV, O’Brien KL, Watt JP (2005). Epidemiology of invasive Haemophilus influenzae type a disease among Navajo and White Mountain apache children, 1988–2003. Clin Infect Dis.

[3] Bolze A, Mahlaoui N, Byun M (2013). Ribosomal protein SA haploinsufficiency in humans with isolated congenital asplenia. Science.

[4] Dennehy PH (2001). Active immunization in the United States: developments over the past decade. Clin Microbiol Rev.

[5] Pavlik DF, Johnston JJ, Eldredge JD, Dehority W (2017). Non-type b Haemophilus influenzae septic arthritis in children. J Pediatr Infect Dis Soc.

[6] Bruce MG, Zulz T, DeByle C (2013). Haemophilus influenzae serotype a invasive disease, Alaska, USA, 1983-2011. Emerg Infect Dis.

[7] Tsang RSW, Ulanova M (2017). The changing epidemiology of invasive Haemophilus influenzae disease: emergence and global presence of serotype a strains that may require a new vaccine for control. Vaccine.

[8] Triden L, Como-Sabetti K, Danila R, et al. Increase of invasive *Haemophilus influenzae* cases and *Haemophilus influenzae* serotype a cases in Minnesota, 2005–2014 [abstract]. Emerg Infect Dis. 2015:292. Board 345. Available at https://wwwnc.cdc.gov/eid/pdfs/ICEID2015.pdf.

[9] Waldman JD, Rosenthal A, Smith AL (1977). Sepsis and congenital asplenia. J Pediatr.

[10] Iijima S (2017). Sporadic isolated congenital asplenia with fulminant pneumococcal meningitis: a case report and updated literature review. BMC Infect Dis.

[11] Gilbert B, Menetrey C, Belin V (2002). Familial isolated congenital asplenia: a rare, frequently hereditary dominant condition, often detected too late as a cause of overwhelming pneumococcal sepsis. Report of a new case and review of 31 others. Eur J Pediatr.

[12] Scheuerman O, Bar-Sever Z, Hoffer V (2014). Functional hyposplenism is an important and underdiagnosed immunodeficiency condition in children. Acta Pediatrica.

[13] Headrick A, Schmit EO, Kimberlin DW (2018). Fulminant Haemophilus influenzae type a infection in a 4-year-old with previously undiagnosed asplenic heterotaxy. Pediatr Infect Dis J.

[14] Berndsen MR, Erlendsdóttir H, Gottfredsson M (2012). Evolving epidemiology of invasive Haemophilus infections in the post-vaccination era: results from a long-term population-based study. Clin Microbiol Infect.

[15] Jin Z, Romero-Steiner S, Carlone GM (2007). *Haemophilus influenzae* type a infection and its prevention. Infect Immun.

[16] Swift AJ, Moxon ER, Zwahlen A, Winkelstein JA (1991). Complement-mediated serum activities against genetically defined capsular transformants of Haemophilus influenzae. Microb Pathog.

[17] Tsang RS, Proulx JF, Hayden K (2017). Characteristics of invasive Haemophilus influenzae serotype a (Hia) from Nunavik, Canada and comparison with Hia strains in other north American Arctic regions. Int J Infect Dis.

[18] Cox AD, Williams D, Cairns C (2017). Investigating the candidacy of a capsular polysaccharide-based glycoconjugate as a vaccine to combat Haemophilus influenzae type a disease: a solution for an unmet public health need. Vaccine.

